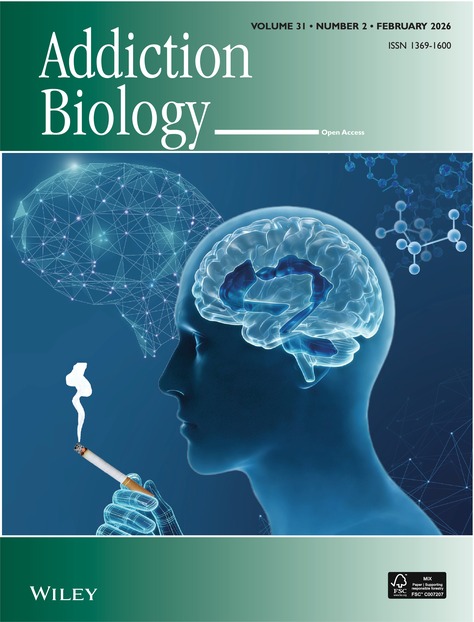# Cover Image

**DOI:** 10.1111/adb.70133

**Published:** 2026-02-25

**Authors:** Zhenzhen Mai, Dahua Yu, Gengdi Huang, Xiaojiao Li, Xuwen Wang, Fang Dong, Yongxin Cheng, Juan Wang, Yuxin Ma, Lin Luo, Kai Yuan, Ting Xue

## Abstract

The cover image is based on the article *Altered Topological Properties of White‐Matter Functional Networks in Young Smokers* by Dahua Yu et al., https://doi.org/10.1111/10.1111/adb.70125.